# Phosphoinositide 3-Kinase Signaling in the Tumor Microenvironment: What Do We Need to Consider When Treating Chronic Lymphocytic Leukemia With PI3K Inhibitors?

**DOI:** 10.3389/fimmu.2020.595818

**Published:** 2021-01-20

**Authors:** Ebru Aydin, Sebastian Faehling, Mariam Saleh, Laura Llaó Cid, Martina Seiffert, Philipp M. Roessner

**Affiliations:** ^1^Molecular Genetics, German Cancer Research Center (DKFZ), Heidelberg, Germany; ^2^Institute of Clinical Sciences, University of Gothenburg, Gothenburg, Sweden; ^3^Medical Faculty, University of Heidelberg, Heidelberg, Germany; ^4^Faculty of Molecular Medicine, Ulm University, Ulm, Germany; ^5^Faculty of Bioscience, University of Heidelberg, Heidelberg, Germany

**Keywords:** phosphoinositide 3-kinase (PI3K), chronic lymphocytic leukemia, tumor microenvironment, idelalisib, phosphoinositide 3-kinase (PI3K) inhibition

## Abstract

Phosphoinositide 3-kinases (PI3Ks) and their downstream proteins constitute a signaling pathway that is involved in both normal cell growth and malignant transformation of cells. Under physiological conditions, PI3K signaling regulates various cellular functions such as apoptosis, survival, proliferation, and growth, depending on the extracellular signals. A deterioration of these extracellular signals caused by mutational damage in oncogenes or growth factor receptors may result in hyperactivation of this signaling cascade, which is recognized as a hallmark of cancer. Although higher activation of PI3K pathway is common in many types of cancer, it has been therapeutically targeted for the first time in chronic lymphocytic leukemia (CLL), demonstrating its significance in B-cell receptor (BCR) signaling and malignant B-cell expansion. The biological activity of the PI3K pathway is not only limited to cancer cells but is also crucial for many components of the tumor microenvironment, as PI3K signaling regulates cytokine responses, and ensures the development and function of immune cells. Therefore, the success or failure of the PI3K inhibition is strongly related to microenvironmental stimuli. In this review, we outline the impacts of PI3K inhibition on the tumor microenvironment with a specific focus on CLL. Acknowledging the effects of PI3K inhibitor-based therapies on the tumor microenvironment in CLL can serve as a rationale for improved drug development, explain treatment-associated adverse events, and suggest novel combinatory treatment strategies in CLL.

## Phosphoinositide 3-Kinase Signaling Pathway

**Figure 1:**
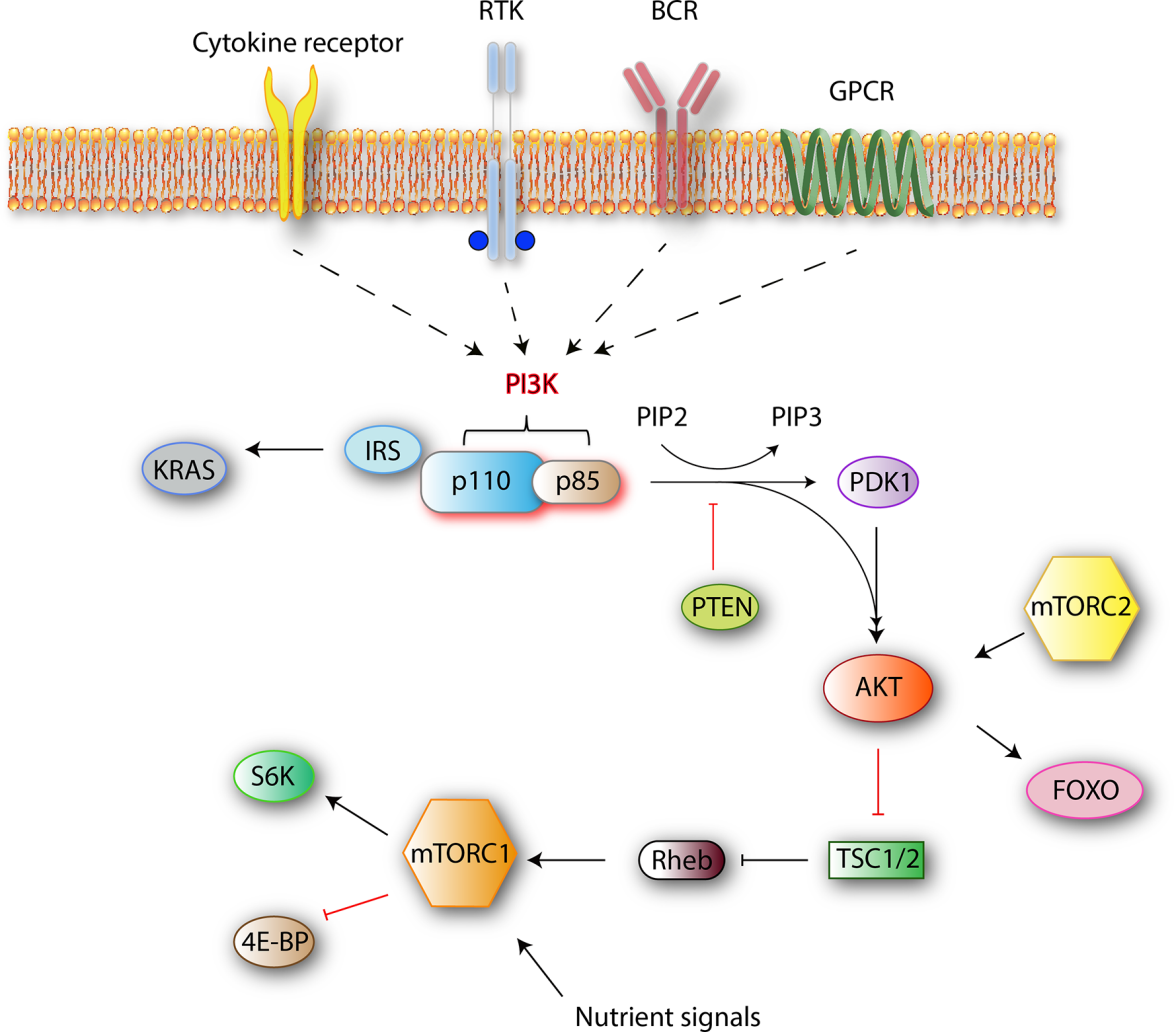
Class I PI3K signaling pathway simplified. Class I PI3Ks reside as dimers in the cells. After receiving an external stimulus, the regulatory p85 subunit interacts with the intracellular section of the activated receptor. The activation of a catalytic p110 isoform triggers a lipid membrane-associated cascade of phosphorylations (PIP2 to PIP3). The PI3K pathway is mediated mainly by PIP3, which is a secondary messenger that acts as a docking site. In the PI3K-AKT pathway, PIP3 can bind to both downstream effector proteins PDK1 and AKT. AKT modification activates the mammalian target of rapamycin complex 1 (mTORC1) by direct phosphorylation, which results in synthesis of growth-, proliferation-, and survival-related proteins (1).

The phosphoinositide 3-kinase (PI3K) family mediates nutrient sensing and metabolic control to prevent cell growth and proliferation in conditions of energy or nutrient deficiency. Several roles are ascribed to PI3K signaling both in normal cell function and cancer. The four best-characterized pathways that activate PI3K are cytokine/chemokine receptors, receptor tyrosine kinases (RTK), B-cell receptor (BCR), and G-protein coupled receptors (GPCR) (**Figure 1**). Stimulation of these receptors causes the autophosphorylation of tyrosine residues on immunoreceptor tyrosine-based activation motifs (ITAMs) that are important mediators of signal transduction in immune cells, leading to the activation of a PI3K p110 catalytic isoform.

PI3Ks phosphorylate the 3-position hydroxy group of the phosphatidylinositol ring. The resulting products are the secondary messengers phosphatidylinositol_(4,5)_P2 (PIP2) and phosphatidylinositol_(3,4,5)_P3 (PIP3), which trigger important physiological changes in cells through AKT/Protein Kinase B (PKB) phosphorylation and downstream kinases. One of those, mammalian target of rapamycin complex 1 (mTORC1), which is a central mediator of the metabolic control, combines signals from nutrients and PI3K to induce cell growth and proliferation. Major components of this pathway are outlined in **Figure 1**.

**Figure 2:**
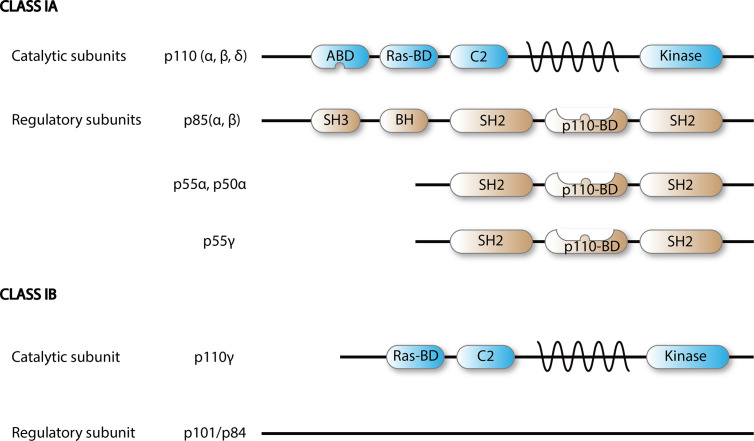
Scheme of PI3K class I isoforms. Functional PI3K is built upon dimerization of a catalytic p110 and a regulatory subunit.

When a ligand binds to RTK, two RTK monomers dimerize, which results in the activation of intracellular tyrosine kinase domain and auto phosphorylation followed by indirect PI3K activation *via* adapter molecules such as the insulin receptor substrate (IRS). Alternatively, BCR-dependent activation of PI3Ks is mediated by Src and Syk family of receptor-associated tyrosine kinases. First, Src-family proteins phosphorylate the tyrosine residues of ITAMs that reside on the cytoplasmic part of the signal transducing subunits of the BCR-associated Ig-α and Ig-β. Phosphorylated ITAMs serve as binding sites for Src-homology 2 (SH2) domain-containing proteins such as B-cell PI3K adaptor protein (BCAP) and CD19 (**Figure 2**). With the help of these proteins, PI3Ks are recruited to the BCR signalosome (2, 3). Next, the regulatory subunit interacts with the intracellular section of the activated receptor *via* its SH2 domain and this event leads to the activation of catalytic p110 isoform which triggers a lipid membrane-associated cascade of phosphorylations (PIP2 to PIP3). The PI3K pathway is mediated mainly by PIP3, which is a secondary messenger that acts as a docking site. In the PI3K-AKT pathway, PIP3 can bind to both downstream effector proteins phosphoinositide dependent kinase (PDK1) and AKT. AKT modification activates the mTORC1 by direct phosphorylation, which results in synthesis of growth, proliferation-, and survival-related proteins (1).

### Isoforms of Phosphoinositide 3-Kinases

There are eight different isoforms of PI3Ks that are grouped into three classes (class I, II, and III) based on their primary structure and regulation. Class I PI3Ks, which this review is focused on, consist of four catalytic isoforms, namely p110α, -β, -γ, and -δ. Class IA PI3Ks are dimers of a p110 catalytic protein and a p85 or p55 regulatory adapter subunit as detailed in **Figure 2** (4). Each regulatory subunit can associate with any of the three catalytic class IA isoforms: p110α, p110β, and p110δ (5).

The expression of distinct catalytic isoforms is cell type specific. P110α and -β are expressed in all cell types. In mice, homozygous knockouts of p110α and p110β are embryonic lethal, emphasizing their physiological importance (6–8). In contrast, p110δ expression is mainly confined to leukocytes (4). Genetically modified mice expressing catalytically inactive PI3Kδ (PI3Kδ^KI^), manifest impaired B-cell, NK cell, and T-cell function (9, 10).

Class IB PI3Ks, which are built by a p110γ catalytic and a p101 regulatory subunit, are selectively expressed by leukocytes (4, 11) and their activation is mediated by GPCRs (4). Knockdown of p110γ in mice causes altered migration and recruitment of myeloid cell populations (12–14), which is in concordance with other reports indicating that the PI3Kγ isoform is expressed mostly in the myeloid cell lineage (15–17).

## Phosphoinositide 3-Kinase Signaling in Cancer and Chronic Lymphocytic Leukemia

### Phosphoinositide 3-Kinase Signaling in Cancer

One of the best-characterized hallmarks of cancer cells is their ability to sustain chronic proliferation (18). While healthy cells can strictly regulate the production and release of growth-promoting signals, cancer cells often fail to do so and malignant transformation occurs as a result of mutations in one or more components of these signaling pathways (18). There are several pathways that influence each other through multifaceted interactions and contribute to tumor development. Some of the major signaling pathways whose components are likely to be cancer drivers include proteins like growth factor receptor tyrosine kinases (e.g., EGFR), lipid kinases (e.g., PI3K), small GTPases (e.g., RAS), oncogenes and tumor suppressors (e.g., MYC, P53), serine/threonine kinases (e.g., RAF, AKT), and cytoplasmic tyrosine kinases (e.g., SRC, ABL) (19–24). Among these, involvement of the PI3K signaling pathway in cancer has been revealed first when PI3K signaling was found to be hyperactive in phosphatase and tensin homologue (PTEN)-deficient tumors (25). Independent of stimulating receptors, PI3Ks can be activated directly *via* oncogenes like *RAS* (26) and their activity is negatively regulated by the tumor suppressor PTEN, that acts as a PI3K phosphatase (**Figure 1**) (27).

Altered PI3K/AKT signaling has been associated with many types of cancer (28). Chronic lymphocytic leukemia (CLL), breast, ovarian, liver, lung cancers, and glioblastomas are well-studied examples in which altered PI3K activity was observed (29–32). Genetic alterations such as amplifications, gain of copy numbers and increased gene expression have been observed for all class I PI3Ks in many different cancer entities (26). P110α (*PIK3CA*) is affected most frequently and a direct oncogenic potential was attributed to it (33), as the signaling of mutant p110α is active and less dependent on external stimuli (34).

### Phosphoinositide 3-Kinase Signaling in Chronic Lymphocytic Leukemia Cells

Compared to the mutation rates in other cancer entities, activating mutations of PI3K are scarce in CLL (35, 36). Genomic analysis revealed an amplification of *PIK3CA* in 3.5% of CLL patients, which is perhaps surprising when all the significant pharmaceutical effort to inhibit the PI3K pathway in this malignancy is considered (37). However, PI3K signaling is constitutively active in CLL cells, even in the absence of genetic drivers (38, 39). There are three well-studied pathways that are capable of activating and maintaining PI3K signaling in CLL cells; BCR, RTKs and cytokine/chemokine receptors (40–42). Although the stimulation of these pathways initiates different cascades of downstream events, they converge at a final point, that is, generation of a domain that can bind to the SH2 region of the regulatory p85 subunit. If the SH2 domain remains occupied, p85 fails to bind a p110 catalytic isoform and inactivate it, which results in constitutively active PI3K (43). The importance of these three pathways varies for different cell types, however, BCR is shown to be the dominant contributor to PI3K activity in B-cell malignancies. Autonomous events or continuous BCR activation by autoantigens have been reasoned for tonic BCR stimulation in CLL (44, 45).

In conclusion, the highly active PI3K signaling pathway in CLL serves as an ideal therapeutic target to inhibit CLL cell proliferation and survival directly.

## Phosphoinositide 3-Kinase Signaling in the Tumor Microenvironment

Although CLL is a genetically heterogeneous disease, it is widely accepted that CLL cells are highly dependent on supporting stimuli of bystander cells in their microenvironment for their survival, proliferation, homing to lymphoid tissues as well as chemo-resistance. Different types of stromal cells, myeloid, NK and T-cells were shown to have dual functions in the tumor microenvironment (TME) of CLL both in patient samples and more recently the Eμ-TCL1 mouse model as reviewed extensively by us and others (46, 47).

In the last 20 years, many mouse models were generated in which different components of the PI3Ks were knocked out (6, 48–51). The results of the studies using these mice need careful interpretation as the alteration of one isoform’s expression can impact on those of other isoforms. As the different catalytic and regulatory subunits have the ability to interact in various combinations (4), the outcome of the expression variations in these knockout models might be far from reflecting the true functions of these proteins. For instance, an increased expression of p85β in addition to decreased expression of the three p110 isoforms (α, β, δ) has been shown in NK cells from p85α^−/−^ mice (5, 52). In another study where p110δ was knocked out, levels of p85α were decreased, p110β and -γ were increased but p110α remained unchanged in NK cells (53). Methodologies involving the generation of point mutations that completely inactivate the catalytic function of isoforms without altering the normal expression levels may provide more reliable conclusions compared to the knockout models that alter the expression of all isoforms, as the former may mimic the normal physiological state better and make it easier to assign functions directly.

### Phosphoinositide 3-Kinase Signaling in Non-Immune Components of the Tumor Microenvironment

In the TME, complex interactions between cancer associated fibroblasts (CAFs), immune cells, signaling molecules, extracellular matrix (ECM), vasculature-associated cells, and malignant cells take place (54). Cancer cells are known to orchestrate the host tissue to facilitate tumor progression and regulate the recruitment of immune cells to the TME. PI3K signaling is engaged in almost all these components, including the stromal compartment. As discussed above, CLL cells require strong support from stromal cells for their proliferation and survival (55). Similarly, Bertrand et al. have shown that stromal cells protect the B-cell acute lymphoblastic leukemia (B-ALL) cells from apoptosis *via* PI3K and MEK pathway dependent mechanisms and co-inhibition of these pathways can synergistically break this apoptotic resistance (56).

The ability of solid tumors to grow depends strictly on oxygen and nutrient delivery through newly developing blood vessels. Accumulating evidence indicates that angiogenesis is also important in hematologic malignancies, including CLL (57). Neovascularization is a cooperative event involving endothelial cells, myeloid cells, and the tumor cells that secrete angiogenic factors such as vascular endothelial growth factor (VEGF). Inhibitors of different PI3K isoforms were shown to generate different vascular responses as the dominant PI3K isoform diverges among these cells (58). Up to date, most of the research that investigates the effect of PI3K inhibition on tumor vascular function was performed with high doses of pan-PI3K inhibitors and these studies are pointing towards a vascular dysfunction which is associated with decreased blood flow and vessel density (59–61). As the anti-angiogenic effect of PI3K inhibition is outcompeted by anti-VEGF therapies (62, 63), PI3K inhibition has not been considered specifically for targeting tumor neoangiogenesis. However, recent preclinical studies show that low doses of PI3K inhibition contribute to vessel normalization which might enable more efficient delivery of chemotherapy drugs and immune infiltration to the tumor sites (64, 65). Overall, these studies support that targeting PI3Ks in tumor and endothelial cells selectively hold the potential to synergize with conventional therapies.

CAFs constitute one of the most abundant but poorly defined cell populations in the TME. A commonly held view is that CAFs promote tumorigenesis *via* remodeling of ECM components, facilitating metastasis, promoting vascularization, and secreting cytokines and growth factors (66). This view is supported also for CLL, where leukemic cells are able to induce the transformation of stromal cells to a CAF-like phenotype by an exosome-mediated fashion (67). In this study, it has been shown that the CLL-derived exosomes are actively taken up by stromal cells both *in vitro* and *in vivo*. Upon uptake, stromal cells gained pro-tumoral characteristics, evident by enhanced proliferation, migration, angiogenesis, and secretion of inflammatory cytokines *via* mechanisms involving activation of AKT and NF-κB. These events are known to be partly involving PI3K signaling (68–70). Thus, it is expected that PI3K inhibition might dampen the pro-tumoral activity of CAFs.

### Phosphoinositide 3-Kinase Signaling Pathway in Immune Cells

All immune cells express receptors that can initiate PI3K signaling, but the components and wiring of the pathway are different in several aspects compared to other cells (1). The major difference is the expression of the catalytic isoform. P110δ and p110γ comprise the dominantly expressed isoforms while p110α and p110β are rarely engaged in immune cells (71). In non-immune cells, a higher degree of PI3K activation usually correlates with an increased downstream function such as apoptosis, survival, proliferation, and growth. However, in immune cells, an increased activation of the PI3K pathway does not necessarily mean a stronger immune reaction. Depending on the cell type, the pathway has the potential to augment or alleviate the response to pathogens as well as tumors. The network of the PI3K pathway downstream of AKT seems to be different for lymphoid cells subsets. MTORC1 phosphorylates a wide range of downstream proteins when stimulated *via* PI3K/AKT and RAS/ERK pathways but it also requires signals delivered through nutrient-sensing pathways to ensure that cells do not intent to proliferate under stress conditions like nutrient deprivation (72, 73). In lymphocytes, mTORC1 was shown to be more dependent on nutrient inputs than on PI3K/AKT activity (74–77). The two best-characterized mTORC1 substrates in mammals are p70 ribosomal S6 kinase 1 (S6K1) and eukaryotic initiation factor 4E-binding protein 1 (4E-BP1) (78). 4E-BP/Eukaryotic translation initiation factor 4E (eIF4E) arm is known to be responsible for regulation of growth and proliferation in lymphocytes (79), whereas in other cell types, S6Ks are the main mediators of cell growth (80). It has also been hypothesized that S6Ks might be involved in lymphocyte differentiation, but to our knowledge this has not been validated yet (81–83) (**Figure 1**). Overall, considering these immune cell-specific features might be useful while estimating the overall impact of PI3K-mTOR inhibition on the TME.

#### Phosphoinositide 3-Kinase Signaling in Myeloid Cells

Innate immune cells such as monocytes, neutrophils, DCs, mast cells, and macrophages are the key components that mediate the initial phase of acute inflammation *via* cytokine/chemokine secretion, extravasation, migration, and phagocytosis. Each of these events requires class I PI3K activation (84). Upon detection of a stimulus, tissue resident macrophages immediately start producing cytokines and chemokines to attract neutrophils and macrophages to the site of infection, which requires p110δ expression (84). These chemoattractants are known to activate neutrophil p110γ through GPCR (14). After reaching the site of inflammation, macrophages and neutrophils phagocyte and lyse the pathogens with nicotinamide adenine dinucleotide phosphate oxidase (NOX)-derived ROS. It has been shown that p110β and p110δ are involved in the regulation of phagocytosis (85–87). In line with this, p110β-deficient mice fail to mediate Fc gamma receptor (FcγR)-dependent activation of neutrophils and produce ROS (88). In addition to initiating ROS production, maintenance of NOX activity for ROS production also requires the activation of p110δ (89).

All class I PI3K catalytic unit isoforms are expressed in myeloid cells. However, current literature suggests that p110δ and p110γ are the critical isoforms for mediating their immune functions. Deficiency of these two isoforms has been reported to induce neutrophilia in mice (90), while inhibition of only one of them did not have a striking effect. Although the p110γ isoform is expressed in many hematopoietic cells, highest level of expression was detected in cells of the myeloid lineage and the inhibition of p110γ resulted in dampened inflammatory responses (14, 91). Of note, neither neutrophil nor macrophage development was impaired in p110γ^−/−^ mice. However, chemoattractant-stimulated neutrophils failed to produce PIP3 and implement respiratory burst (14). Motility and migration mechanisms have also been noted to be defective, both in neutrophils and macrophages (14). Similarly, the migration of antigen-loaded DCs from the tissue to draining lymph nodes was impaired demonstrating the non-redundant functions of p110γ (92).

##### Effect of PI3K Inhibition in Myeloid Cells in Cancer

PI3K signaling mediates an immunosuppressive phenotype in myeloid cells to prevent excessive innate immunity in chronic infections and inflammation (89, 93–95). This phenomenon supported the idea to pharmacologically target the PI3K p110γ isoform with specific inhibitors for the treatment of inflammatory diseases but the possibility of dampening the host defense has slowed down the development of this idea.

However, the pro-inflammatory potential of PI3K inhibitors might provide new opportunities in cancer treatment. In many cancer entities, pro-inflammatory cytokine expression is correlated with better prognosis. High expression levels of p110γ that induces immunosuppression in TAMs, were reported in several studies (15, 96, 97). Selective inhibition of PI3Kγ in mouse models of melanoma and breast cancer revealed a downregulation of M2 markers (*Tgfb1*, *Arg1*, *Ido1*) and an upregulation of M1 markers (*Il12a* and *Nos2*) (98), demonstrating the influence of this isoform on the phenotype and polarization of TAMs. Interestingly, reduced tumor growth as well as enhanced anti-tumoral T-cell responses have been noted upon genetic or pharmacological p110γ inhibition (15, 96, 98). Of note, cancer cells of most solid tumors do not express p110γ and anti-tumoral effects of p110γ inhibition might be attributed to TAMs and the TME.

Recently, the immune microenvironment has gained attention in cancer therapy. Although the approval of checkpoint inhibitors has been a significant breakthrough in immunotherapy, multiple resistance mechanisms exist, and the presence of tumor infiltrating myeloid cells are among these. Following encouraging pre-clinical results, a phase 1 trial combining immune checkpoint blockade and inhibition of p110γ has been initiated, but results are pending (NCT02637531) (98).

Inactivation of p110δ isoforms mostly interferes with the functions of adaptive immune cells but this isoform has also been implicated in controlling the pro-tumorigenic effect of myeloid cells in cancer (99, 100). In one study, histological analysis of patient breast tumors demonstrated a correlation between the disease progression and levels of PI3Kδ in tumor cells. In addition to the tumor cells, CD68^+^ macrophages also manifested strong PI3Kδ staining (101). Treatment with a PI3Kδ-specific inhibitor, IC87114, reduced the volume of human breast tumors in xenograft models when administered intratumorally or orally (101). Intratumoral treatment only slowed down tumor growth, as a small but significant proliferation of tumor cells was still detectable (101). However, oral administration of the inhibitor totally stopped tumor growth and reduced macrophage infiltration (101), suggesting that PI3Kδ inhibition in these cells might be a contributing factor to the anti-tumoral effect. To further evaluate the impact of macrophage PI3Kδ inhibition on tumor growth, the authors adoptively transferred either PI3Kδ-knockout (KO) or wildtype (WT) macrophages to tumor bearing NOD/SCID-gamma null (NSG) mice (101). Tumor growth was significantly reduced in the mice that received PI3Kδ-KO macrophages compared to the ones that received WT macrophages (101). In addition, PI3Kδ-KO macrophages were less competent in infiltrating the tumors compared to WT macrophages (101), demonstrating that inhibition of macrophage PI3Kδ might be sufficient to reduce the infiltration of macrophages to tumors and thereby impact on tumor growth. In the final set of experiments, the researchers inoculated PI3Kδ expressing tumors to macrophage-deficient NSG mice and PI3Kδ-deficient tumors to Balb/c nude mice with functional and PI3Kδ-proficient macrophages. While treatment with the PI3Kδ inhibitor IC87114 reduced tumor growth in both groups, complete abrogation was only seen in mice with PI3Kδ-expressing tumors and WT macrophages (101). Overall, these results suggest that targeting PI3Kδ both in tumor cells and TAMs is likely to be a promising strategy for treatment of breast cancer.

The exact function of PI3K isoforms in DCs is unclear. To study the impact of PI3K inhibition in DCs, Marshall et al. have employed different mouse tumor models and reported that either broad or β-, δ-, or γ-isoform-specific inhibition reduced levels of IL-10 and TGFβ but enhanced IL-12 expression (99). This caused a less immunosuppressive TME and facilitated the priming of Th1 T-cells *in vivo*. An important finding of this study is that selective inhibition of PI3Kβ and -δ isoforms reduced IL-10 and TGFβ expression in toll-like receptor (TLR)-activated DCs pulsed with dead tumor cells *in vitro*. Adoptive transfer of pulsed DCs into tumor-bearing mice in combination with pan-PI3K inhibition resulted in a significant increase in anti-tumoral T-cell response, decrease in tumor growth and prolonged overall survival, demonstrating the potential of PI3K inhibitors for the development of tumor DC-based vaccines.

#### Effect of Phosphoinositide 3-Kinase Inhibition on Myeloid Cells in Chronic Lymphocytic Leukemia

Twenty years ago, Burger et al. discovered that peripheral blood monocytes differentiate in co-culture with CLL cells to adherent cells that support the survival of CLL cells *in vitro* (102). These cells were named as nurse-like cells (NLCs), and only later on, it was understood that they resembled tumor-associated macrophages (TAMs) in solid tumors (103, 104). NLCs exhibit an M2-like phenotype and besides delivering survival signals, they were suggested to have immunosuppressive functions (55, 105).

Research investigating the effect of PI3K inhibition on myeloid cells in CLL is scarce. Thus far, efforts have been dedicated to identifying the effect of clinically approved PI3K inhibitors in modulating the effect of type I anti-CD20 monoclonal antibody (e.g., rituximab and ofatumumab) treatment. More specifically, the role of PI3K in regulating antibody-dependent cellular cytotoxicity (ADCC) and antibody-dependent phagocytosis (ADP) responses has been investigated. Da Roit et al. described an effect of the PI3Kδ inhibitor idelalisib on inhibiting activation of polymorphonuclear neutrophils (PMN) and phagocytosis of anti-CD20 opsonized CLL cells by macrophages (106). Based on these observations, Enya Chen et al. investigated the role of PI3K-p110 in modulating antibody-mediated responses by macrophages in CLL (107). The authors demonstrated that class I PI3K p110 isoforms α, β, γ, and δ are expressed in nurse-like cells (NLCs) of both anti-CD20 antibody-sensitive and -resistant CLL patients (107). The effect of PI3K inhibition on ADCC and ADP was found to be FcγR-mediated and independent of downstream spleen tyrosine kinase (SYK) and Bruton’s tyrosine kinase (BTK) signaling. Inhibition of p110δ, both pharmacologically and *via* siRNA knockdown, decreased ADCC and ADP in CLL-derived NLCs (107), suggesting that this catalytic isoform is essential for CLL-derived NLC as well as macrophage response to therapeutic antibodies.

In a recent study, idelalisib has been shown to prevent AKT-dependent phosphorylation of the p47^phox^ subunit of NOX2, resulting in suppressed rituximab-induced ROS formation in human monocytes (108). In co-cultures of monocytes and NK cells, idelalisib rescued NK cells from ROS-induced toxicity and thus improved NK cell ADCC against primary CLL cells and 221 B-lymphoblastoid cells (108).

In addition to its role in regulating ADCC, the PI3K pathway is also involved in migration and homing of CLL cells which circulate between peripheral blood and secondary lymphoid organs (109). Trafficking of CLL cells is regulated by stromal cells and NLCs in the lymph nodes that secrete chemokines including C-X-C motif chemokine ligand (CXCL)12, CXCL13, C-C motif ligand (CCL)19, and CCL21 (110). Upon the activation of chemokine receptors, PI3K signaling directs the CLL cells towards stromal cells that provide tumor supportive stimuli (111). As expected, inhibition of the PI3K pathway *in vitro* with PI3Kδ-specific idelalisib was shown to result in impaired homing of CLL cells to stromal cells, due to their reduced response to CXCL12 and CXCL13 (109). These findings partly explain the rapid lymph node shrinkage in CLL patients treated with PI3Kδ inhibitor idelalisib (112).

**Figure 3:**
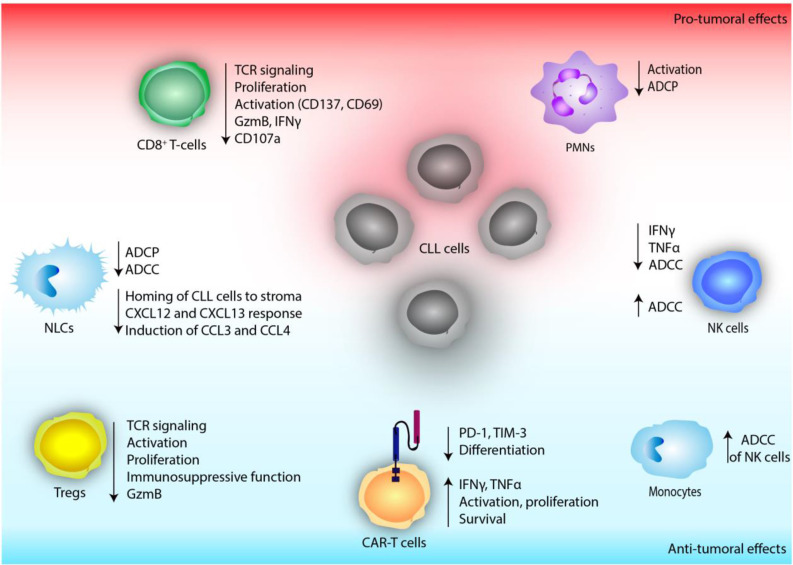
The effect of defective PI3K signaling on immune cells of the tumor microenvironment in chronic lymphocytic leukemia (CLL).

Overall, current research highlights the importance of PI3K activity in immune responses to therapeutic antibodies and suggests that both enhancing and inhibiting this pathway might improve these responses (**Figure 3**). Nevertheless, further *in vitro* and *in vivo* studies are needed to unravel the diverse and complex effects of PI3K inhibition in the myeloid compartment of CLL.

#### Phosphoinositide 3-Kinase Signaling in Natural Killer Cells

Information about how NK cells are affected by PI3K inhibition is relatively sparse and derived mainly from knockouts of PI3K isoforms in mice. NK cells are critical mediators of innate immunity that do not require prior sensitization or antigenic stimulation. Upon activation by tumor cells, they can exert their cytolytic functions *via* granzyme (Gzm)- or receptor-mediated fashion (113, 114). They are also producers of an array of cytokines including interferon gamma (IFNγ) and tumor necrosis factor alpha (TNFα), which act on other immune cells to enhance the immune response. There are several studies providing evidence that PI3K isoforms control signaling checkpoints in cytokine expression and secretion in mouse NK cells (115). PI3Ks are involved in the signaling downstream of different NK cell activation receptors such as NKG2D, CD16, and CD28. In addition, PI3K–mTOR pathway is indispensable for efficient NK cell activity, specifically for cytokine responses in IL-15 primed NK cells (116). PI3K inhibitors were also shown to have an impact on NK cell granule polarization. Upon tumor contact, NK cells start to mobilize perforins and GzmB towards the tumor cells (117). Zhong et al. have shown that pre-treatment of NK cells with pan-PI3K inhibitors (wortmannin or LY294002) effectively stopped this intracellular polarization of cytotoxic granules (118). A complementary study has also shown that mice lacking the regulatory p85α subunit and its alternatively spliced variants (p55α/p50α) had impaired lineage commitment leading to reduced NK cell numbers in the bone marrow and liver. In addition to genetic inhibition, broad pharmacological PI3K inhibition with wortmannin also reduced the cytotoxicity against EL-4 lymphoma cells and cytokine/chemokine generation was significantly compromised (119). Other studies that support these findings exist, in which treatment with different PI3K inhibitors (wortmannin, LY294002, IPI-145) quenched mouse and human NK cell cytotoxicity against tumor cells (120–122) while inhibition of a single p110 isoform did not have a profound effect. This phenomenon brings up the question whether different p110 isoforms can compensate for/replace each other in NK cells. Kim et al. have investigated the relative contribution of p110δ and -γ isoforms on NK cell anti-tumor cytotoxicity and provided an answer to this question to some extent (123). They reported a reduction in NK cell numbers and production of the cytokines IFNγ, TNFα, and granulocyte-macrophage colony-stimulating factor (GM-CSF) in p110δ-deficient, but not p110γ-deficient NK cells. When both p110δ and p110γ isoforms were inactivated by a combination of genetic and biochemical approaches, NK cell cytotoxicity was attenuated, which was not observed upon inhibition of a single isoform (123). Therefore, it is likely that cooperation of isoforms can compensate for the maintenance of cytotoxic function in NK cells. However, the overall results suggest that p110δ is the dominant PI3K isoform for cytokine secretion by NK cells. In line with this study, Saudemont et al., have also proposed that P110δ is the dominantly expressed and preferentially activated isoform in NK cells (124).

Using a murine model of catalytically inactive p110δ (PI3Kδ^KI^), Guo et al. have shown that p110δ plays a crucial role in NK cell terminal maturation and cytokine/chemokine generation (10). Moreover, the PI3Kδ^KI^ mice had reduced NK cell numbers and maturation. NK-mediated cytotoxicity was slightly impaired against EL-4, YAC-1, and RMA-S tumor cells *in vitro* and *in vivo*. However, cytokine and chemokine generation by NK cells was severely affected accompanied by a reduced c-Jun N-terminal kinase (JNK) 1/2 phosphorylation in response to NKG2D-mediated activation. The study provides evidence for the role of PI3Kδ in downstream JNK activation that regulates NK cell cytokine generation (10).

Another study using the same mouse model showed that the NK cells derived from PI3Kδ^KI^ mice showed comparable cytotoxicity to WT NK cells, suggesting that p110δ is not indispensable to kill RMA-S lymphoma cells *in vitro* (115). On the contrary, mice lacking a functional p110δ failed to eliminate *i.p.* administered syngeneic tumor cells. The authors hypothesized that the failure might be specific to the intraperitoneal model they have used, or it may possibly indicate a defect of NK cell migration to the tumor site (115). Two years later, the same group has tested whether the p110δ isoform was essential for NK cell trafficking. Using genetically modified mice, they observed that p110δ was indispensable for chemotaxis to sphingosine-1-phosphate (S1P) and CXCL10, NK cell distribution throughout lymphoid and non-lymphoid tissues and for extravasation to tumors (124).

#### Effect of Phosphoinositide 3-Kinase Inhibition on Natural Killer Cells in Chronic Lymphocytic Leukemia

NK cells are not able to kill CLL cells directly, however they contribute substantially to ADCC (125, 126). Rituximab, a chimeric monoclonal antibody that targets CD20, was approved in 2010 and served as standard therapy in combination with chemotherapy for the treatment of CLL. Fc region of the antibody communicates with the NK cell surface Fc-γ-receptor CD16 (FcγRIIIA) (127). Fc receptor ligation leads to PI3K activation in NK cells, triggering release of cytotoxic granules and ADCC (123, 128). Earlier *in vitro* studies using idelalisib have not reported any significant cytotoxicity towards NK cells (38). Interestingly, pre-treatment of NK cells with idelalisib inhibited their production of inflammatory cytokines, such as TNFα and IFNγ but did not alter their ADCC against CLL cells (38) (**Figure 3**).

Considering that the ADCC of NK cells is mediated through PI3K activation, it might be expected that inhibition of PI3Kδ would limit the clinical efficacy of rituximab. For clinical use, idelalisib is only approved in the combination with αCD20 antibodies and this treatment is efficacious (129). To investigate whether idelalisib dampens NK cell mediated ADCC, a direct comparison of idelalisib *versus* idelalisib + rituximab would be necessary.

### Phosphoinositide 3-Kinase Signaling in T-Cells

PI3K signaling is involved in several T-cell specific functions spanning from their activation to apoptosis. The activity of PI3K is regulated by the co-stimulatory receptors CD28 (4, 9) and inducible T-cell co-stimulator (ICOS) (4). Activation of these receptors results in subsequent activation of PI3Kδ, the dominantly expressed PI3K isoform, which leads to proliferation and differentiation into regulatory, effector, and memory T-cell phenotypes (4, 9). In addition, PI3Ks regulate survival and apoptosis by increase of B-cell lymphoma-extra large (BCL-XL) expression or IL-2 secretion in response to T-cell receptor (TCR) activation (4). These functions of T-cells involve calcium flux, in which PI3Ks are also involved (4). Resting T-cells sustain a low level of Ca^2+^. Upon TCR activation, the cells experience an influx of Ca^2+^ (130). It is reported that PI3Ks are involved in the regulation of calcium signaling *via* IL-2-inducible T-cell kinase (ITK), indicating the significance of the crosstalk between these pathways (4).

#### Effect of Phosphoinositide 3-Kinase δ Inhibition on T-Cells

PI3Kδ is crucial for the function of T-cells, as reduced activity of PI3Kδ has been shown to diminish TCR signaling of murine (9, 131–134) as well as human T-cells (135–137) *in vitro*, including all major CD4^+^ and CD8^+^ T-cell subsets. Subsequently, reduced proliferation of murine (9, 132, 133, 135, 137, 138) as well as human T-cells (134–137) has been observed upon abrogated PI3Kδ signaling, without induction of apoptosis (133). Of note, some combinatory approaches targeting the TCR as well as the co-stimulatory receptor CD28 or supplying IL-2 have been shown to overcome the negative effects of PI3Kδ inhibition on T-cell activation (9, 133, 135).

T-cells differentiate after the activation of their TCR into antigen-experienced effector and memory cells (139, 140). During this process, surface marker expression as well as transcriptional programs change, which for example enables effector and memory T-cells to home to distinct organs. Memory T-cells are equipped to provide fast cytotoxic functions and proliferation upon secondary recall infections (140, 141). In line with reduced TCR-activation, impaired PI3Kδ signaling has been shown to result in a higher expression of CD62L (138, 142, 143), CD127 (143) as well as CCR7 (9, 143), and therefore, less terminally differentiated T-cells. Long-term culture of T-cells using repeated TCR stimulations in the presence of PI3Kδ inhibitors resulted in higher proliferation rates and IL-2 secretion (138), likely caused by initial blockade of T-cell activation and differentiation. The typical memory T-cell markers such as CD62L or CCR7 are important mediators of migration and homing. In contrast to the higher expression of these markers in PI3Kδ-defective T-cells, transfer of WT, or PI3Kδ^KI^ T-cells into syngeneic hosts did result in comparable homing to lymph nodes (133), suggesting that the expression of naïve/memory markers does not affect the migration of these cells.

Following the clonal expansion of T-cells, particularly their production of effector molecules is essential to control pathogens as well as cancer. After *in vitro* or *in vivo* stimulation of PI3Kδ-defective T-cells, a lower cytokine production of IFNγ, IL-2, IL-17, and TNFα (135) has been observed compared to WT cells. This was confirmed using an antigen-specific stimulation of T-cells of WT or PI3Kδ^KI^ mice, in which a reduced expansion as well as IFNγ production of mutant T-cells was noted (133).

In conclusion, PI3Kδ is essential for TCR signaling and subsequent activation, proliferation, and cytokine production of T-cells. However, it is likely that the negative effects of PI3Kδ inhibition in T-cells can be partially overcome by applying strong or combinatory stimuli.

##### Effect of Phosphoinositide 3-Kinase δ Inhibition on CD4^+^ T-Cells

CD4^+^ helper T-cells (Th cells) are involved in multiple immune functions, such as control of immune reactions by Tregs as well as providing help in the clearance of pathogens by Th cells (46).

IFNγ, IL-4 and IL-17 are the key cytokines of Th1-, Th2-, and Th17-mediated responses, respectively (46). Following *in vitro* stimulation in the presence of PI3Kδ inhibitors, a reduced production of IFNγ (133, 135), IL-4 (133), and IL-17 have been noted (135). Of interest, memory CD4^+^ T-cells were more susceptible to abrogated PI3Kδ signaling than their naïve counterparts (135). These data highlight that PI3Kδ inhibition affects the function of Th cell subsets and suggest that antigen-experienced T-cell subsets might be more dependent on PI3Kδ signaling than naïve T-cells.

Tregs are crucial in maintaining orchestrated immune reactions by paracrine secretion of IL-10 or direct, contact-mediated inhibition of effector responses (46). In line with the reduced proliferation of this cell type upon PI3Kδ inhibition *in vitro* (136, 137), lower frequencies of Tregs in lymphoid organs and blood of p110δ^KI^ mice have been observed (100, 144), and shown to be most likely due to an increased rate of apoptosis (137). In line with reduced TCR activation upon PI3Kδ inhibition, lower expression levels of FOXP3, CD25, ICOS, and PD-1 have been noted in human Tregs *in vitro* (136), indicating a reduced activation state and immunosuppressive potential of this cell type. Accordingly, PI3Kδ-inhibited murine (144) as well as human Tregs (136) were reported to be less efficient in suppressing effector T-cell proliferation *in vitro*, and lower concentrations of the Treg-specific cytokine IL-10 but higher concentrations of the effector cytokines IL-2 and TNFα have been observed in the culture supernatants upon PI3Kδ inhibition (136). Interestingly, the negative effect of PI3Kδ inhibition could be overcome upon co-culture with antigen-presenting cells (APCs) (144), which could provide strong co-stimulatory signals to compensate for the inhibitory effects of abrogated PI3Kδ signaling. Comparing the sensitivity of Tregs and effector T-cell populations to PI3Kδ inhibition, *in vitro* assays showed that Tregs seem to be more sensitive (134, 136, 137). In sum, the functions of helper T-cells as well as Tregs are dependent on PI3Kδ signaling. Moreover, it is likely that Tregs are more sensitive to PI3Kδ inhibition than effector T-cells, which could unleash immunosuppressive bonds from effector cells and enhance their effector function.

##### Effect of Phosphoinositide 3-Kinase δ Inhibition on CD8^+^ T-Cells

CD8^+^ T-cells are important for the control of pathogens as well as anti-tumoral immunity (46). In addition to the previously mentioned reduced *in vitro* proliferation of T-cells, the numbers of pathogen-specific CD8^+^ T-cells was also reduced in PI3Kδ-deficient mouse models of infections (131, 132). Therefore, a thorough analysis of CD8^+^ T-cell function upon PI3Kδ inhibition is essential, as enhanced effector functions could compensate for the reduced expansion of these cells and result in similar immunological properties.

The expression of early activation markers is often used to analyze TCR signaling activity and the activation status of T-cells. Upon TCR stimulation reduced expression of the activation markers CD25 (132), CD69 (132, 134, 143), killer cell lectin-like receptor subfamily G member 1 (KLRG1) (143) as well as PD-1 (143) have been noted in murine, PI3Kδ-inhibited CD8^+^ T-cells compared to untreated cells. Similarly, abrogated PI3Kδ signaling resulted in a reduced expression of CD69 (134, 135) as well as T-cell immunoglobulin and mucin-domain containing-3 (TIM-3) (143), a marker of activation-induced dysfunction, on human CD8^+^ T-cells after TCR engagement in comparison to PI3Kδ-proficient T-cells, suggesting that not only numbers, but also activation of CD8^+^ T-cells could be altered by PI3Kδ inhibitors.

Once activated, CD8^+^ T-cells secrete granzymes and cytokines such as IFNγ or TNFα, which is essential for their immune control of pathogens and cancer (46). *In vitro* analysis of CD8^+^ T-cell function after antigen-specific or antibody-mediated TCR stimulation revealed lower expression of the effector molecules IFNγ (131, 135), TNFα (132), GzmB (100, 131), GzmA (100, 142), perforin (100), and IL-2 (135) upon PI3Kδ inhibition.

In summary, PI3Kδ is essential for the activation and cytotoxic function of CD8^+^ T-cells, which could in turn result in a reduced immune surveillance and anti-tumoral immunity in individuals that are treated with PI3Kδ inhibitors.

#### Targeting Phosphoinositide 3-Kinase δ in T-Cells in Cancer

The function of PI3Kδ in T-cells has extensively been investigated in many murine cancer models. According to previously mentioned results, global PI3Kδ abrogation in mice caused a reduced frequency and absolute number of Tregs at tumor sites (137, 142, 145) and in draining lymph nodes (100). Of interest, reduced frequencies of Tregs have already been found in spleens of mice as early as 3 days after a single dose of idelalisib (137), suggesting a very fast reaction to PI3Kδ inhibition. Concomitantly with reduced Treg numbers, improved tumor control has been noted in various tumor models using either pharmacological or genetic inhibition of PI3Kδ (100, 142, 145). Utilizing FoxP3^Cre^ x δ^flox/flox^ mice, in which PI3Kδ-deficiency is specific to Tregs, a reduced tumor growth and improved survival was noted in comparison to systemic PI3Kδ-deficient or PI3Kδ-proficient mice (100). The improved tumor control in the presence of PI3Kδ-deficient Tregs was further confirmed by transfer of WT or PI3Kδ^KI^ Tregs into PI3Kδ^KI^-mice (100). This data highlight that inhibition of Treg-PI3Kδ reduces the accumulation and immunosuppressive function of Tregs which results in an enhanced anti-tumoral immunity in mouse models of cancer.

Supported by reduced Treg functions upon PI3Kδ inhibition, enhanced tumor-infiltration, and cytotoxic function of CD8^+^ T-cells were expected. Accordingly, systemic PI3Kδ inhibition resulted in an increased ratio of CD8^+^ T-cells to Tregs and increased numbers of CD8^+^ tumor-infiltrating lymphocytes (TIL) in mouse models of carcinoma (100, 145). Further, transfer of WT but not PI3Kδ^KI^ Tregs into PI3Kδ^KI^ mice resulted in reduced infiltration of CD8^+^ TILs (100). These data strongly support the hypothesis that reduced Treg function, caused by defective PI3Kδ signaling, increases TIL infiltration and enhances anti-tumoral immunity.

Seminal work by Lim et al. investigated several tumor models expressing the ovalbumin (OVA) antigen, namely EL-4 lymphoma, MC-38 colon, and LLC lung carcinoma (142). For EL-4 and MC-38 cancer models, tumor growth was dependent on Treg-PI3Kδ, as depletion of Tregs using FoxP3^DTR^ mice, as well as Treg-specific, genetic PI3Kδ deficiency resulted in a reduced tumor burden (142). In line, transfer of WT or PI3Kδ^KI^, tumor-specific CD8^+^ T-cells into EL-4 tumor-bearing mice did not impact on the disease progression (100). This data suggest that CD8^+^ T-cells are not sufficient to mediate disease control in the EL4 model while targeting PI3Kδ in Tregs has a high potential.

In contrast, growth of LLC- and MC-38 tumors was neither altered by depletion of Tregs, nor using a systemic PI3Kδ^KI^ model (142), suggesting that tumor control in these models relies at least partially on functional PI3Kδ in other immune cells, likely CD8^+^ T-cells. In line, LLC tumor cells were most susceptible to CD8^+^ T-cell mediated killing *in vitro*, followed by MC-38 and EL-4 tumors (142). This anti-tumoral immunity was abolished if CD8^+^ T-cells harbored a PI3Kδ^KI^ mutation (142). Similarly, *ex vivo* analysis of CD8^+^ TILs of LLC-OVA or MC-38 tumors revealed a reduced expression of the cytotoxic molecules GzmA and GzmB if PI3Kδ-signaling was abrogated (142).

In conclusion, the effects of PI3Kδ inhibition on T-cells and subsequently on tumor control are highly contextual. In tumors that are controlled by an immunosuppressive, Treg-dependent microenvironment, PI3Kδ inhibition can succumb the pro-tumoral TME and result in slowed tumor progression. On the contrary, in tumors that are controlled by CD8^+^ T-cells, PI3Kδ inhibition reduces the tumor infiltration, activity as well as tumor control of this cell type. Of note, even though PI3Kδ deficiency does not have a significant impact on tumors that are resistant to CD8^+^ T-cell mediated killing, it is likely that abolished CD8^+^ T-cell function could cause a reduced response to pathogens and therefore, limit the success of combining immunotherapeutic treatment approaches and PI3Kδ inhibitors.

#### Effects of Phosphoinositide 3-Kinase δ Inhibition on T-Cells in Chronic Lymphocytic Leukemia

T-cells, their subsets and function are profoundly altered in CLL and known to be of pathological relevance, as recently reviewed by us (46), emphasizing the need to evaluate the effect of PI3K inhibitors on these cells.

Similar as in the above-mentioned tumor models, PI3Kδ inhibition by idelalisib treatment of CLL patient-derived T-cells *in vitro* also resulted in reduced proliferation, expression of activation markers (CD69, CD25) (146), and cytokine production, including IL-2, TNFα, and IFNγ (146). Using Boyden-chamber migration assays, a reduced migration of idelalisib pre-treated T-cells obtained from CLL patients has been observed (146). This data highlight that idelalisib pre-treatment reduces the function of CLL patient-derived T-cells, emphasizing the need for more detailed investigations.

##### Effect of Phosphoinositide 3-Kinase δ Inhibition on Tregs in Chronic Lymphocytic Leukemia

Tregs have repeatedly been reported to be more abundant in CLL patients than healthy individuals, as recently reviewed by us (46). As expected, PI3Kδ inhibition decreased TCR signaling of Tregs, which were isolated from TCL1-leukemia bearing mice, in a dose-dependent manner (134). Accordingly, reduced proliferation and numbers of Tregs have been noted *in vivo* using either pharmacological PI3Kδ inhibition in the TCL1 AT model of CLL, in which leukemic splenocytes of Eµ-TCL1 mice were transplanted into syngeneic WT mice (134) or PI3Kδ^KI^ x Eµ-TCL1 mice (147). Functionally, PI3Kδ inhibition reduced the expression of activation markers and their immunosuppressive function, as a reduced secretion of GzmB was noted (134). These findings indicate that preclinical PI3Kδ inhibition in CLL results not only in defects of Treg proliferation but also induces their reduced activation and immunosuppressive phenotype.

Until now, reports analyzing Tregs in samples of CLL patients undergoing idelalisib treatment are scarce. Comparison of blood samples before idelalisib treatment initiation and during the treatment revealed a reduced frequency of circulating Tregs (148) as well as a reduced fraction of KI-67^+^, proliferating Tregs, in comparison to pre-treatment samples (136), implicating that idelalisib treatment of CLL patients likely reduces accumulation and proliferation of Tregs (**Figure 3**). Although effects of reduced CLL burden cannot be excluded to contribute to these observations, these results are concordant with the findings of other pre-clinical models of different cancer entities.

##### Effect of Phosphoinositide 3-Kinase δ Inhibition on CD8^+^ T-Cells in Chronic Lymphocytic Leukemia

In CLL, CD8^+^ T-cells have been proposed to contribute to the control of disease progression (46, 149), thus, their function has to be maintained during treatment. As suggested by other models, PI3Kδ inhibition *ex vivo* decreased TCR signaling of CD8^+^ T-cells, which were isolated from TCL1-leukemia bearing mice, in a dose-dependent manner (134). And a reduced proliferation of CD8^+^ T-cells has been observed after PI3Kδ inhibitor treatment *in vivo* in the preclinical TCL1 AT model of CLL (134) as well as in another model, in which OVA-expressing TCL1 leukemic cells were transplanted into PI3Kδ^KI^ mice (147). Concomitantly with their reduced TCR signaling activity and proliferation upon defective PI3Kδ signaling, a reduced expression of the activation markers CD137 and CD69 and an enrichment of naïve CD8^+^ T-cells have been noted in the TCL1 AT model (134). Functionally, PI3Kδ inhibition caused a reduced effector molecule production of CD8^+^ T-cells, as less IFNγ, GzmB, and CD107a were detected, even if focusing the analysis on the antigen-experienced effector population (134). In conclusion, preclinical PI3Kδ inhibition in the TCL1 AT mouse model of CLL reduces the proliferation, expression of activation markers, differentiation, as well as effector function of CD8^+^ T-cells, which likely contributes to a diminished CLL-control by this cell type.

In contrast to these results, Dong et al. performed a transplantation study of TCL1 leukemic cells into PI3Kδ^KI^ mice, which resulted in reduced TCL1 leukemia growth in comparison to WT recipient mice (147). Of importance, tumor cell transplantation in mice with genetic differences between the donor and recipient has been shown to cause tumor rejection in the TCL1 mouse model of CLL (150) as well as a model of multiple myeloma (151), which seems a likely explanation for the lower TCL1 tumor burden in PI3Kδ^KI^ *versus* WT mice. Re-challenge of these PI3Kδ^KI^ mice, which previously rejected TCL1-leukemia, with tumor cells resulted again in tumor rejection and expansion of CD44^+^ effector/memory CD4^+^ as well as CD8^+^ T-cells (147). Rather than providing evidence on the role of PI3Kδ for CD8^+^ T-cell function in CLL, these results suggest that PI3Kδ^KI^ mice are still proficient in a recall, memory T-cell response. Moreover, graft-rejection of TCL1-leukemia is based on a strong T-cell stimulus which potentially overcomes inhibitory effects of abrogated PI3Kδ signaling.

Most evidence about the impact of PI3Kδ inhibition on CD8^+^ T-cells in CLL patients is derived from preclinical studies. In the clinical study by Chellappa et al. analysis of blood samples of idelalisib-treated CLL patient showed a reduced frequency of KI-67 expressing, proliferating CD8^+^ effector T-cells during idelalisib treatment for two out of three patients (136), suggesting that PI3Kδ inhibition might not be beneficial for CD8^+^ T-cell mediated tumor control as well as their clearance of pathogens. But based on the very small patient cohort in this study and recent findings showing that CD8^+^ T-cells derived from lymphoid organs of CLL patients are more active than their blood-derived counterparts (149, 152), the provided analysis of blood T-cells during idelalisib-treatment allows only for very limited conclusions.

In summary, although there is evidence suggesting that pre-clinical as well as clinical PI3Kδ inhibition in CLL diminishes CD8^+^ T-cell function (**Figure 3**), further studies investigating patient samples are needed to fully elucidate the effects of inhibited PI3Kδ signaling on CD8^+^ T-cells in CLL.

#### Impact of Phosphoinositide 3-Kinase δ Inhibition on Adoptive Cell Transfers and Chimeric Antigen Receptor T-Cell Therapy in Chronic Lymphocytic Leukemia

Adoptive cell transfer-based therapies such as chimeric antigen receptor (CAR) T-cell therapy, are new approaches to specifically target tumor cells that are currently also investigated in CLL, as recently reviewed by us (46). So far, the effect of PI3Kδ inhibition on T-cells during the generation of the infusion T-cell product on anti-tumor activity has been investigated in different cancer entities, including CLL. Transplantation of PI3Kδ inhibitor pre-treated CD8^+^ T-cells into B16 melanoma-bearing WT mice, resulted in a better tumor control (138, 143), which could be further enhanced by vaccination with the tumor antigen (138). Similarly to beforehand mentioned, *in vitro* PI3Kδ inhibition results in a less differentiated phenotype of T-cells (143) and in increased proliferation rates of T-cells after long-term cultivation (138). Accordingly, the frequency of PI3Kδ inhibitor pre-treated T-cells within the tumor site trended to be higher in comparison to vehicle-treated controls (143). Of interest, transfer of naïve T-cells similarly reduced the tumor size as seen for PI3Kδ inhibitor pre-treated CD8^+^ T-cells (143). This data suggest that PI3Kδ inhibition during the generation of the T-cell product results in their reduced differentiation, activation, and subsequently higher proliferation *in vivo* as well as a better tumor control.

In comparison to other B-cell malignancies, the remission rates of CD19 CAR T-cell therapies in CLL have been disappointing (46). Therefore, different efforts were taken to improve treatment efficacy. Among them, pre-treatment of CAR T-cells with ibrutinib (153) or idelalisib (154) have been explored. PI3Kδ inhibition during CAR T-cell production of patient derived T-cells resulted in lower proliferation rates of CD4^+^ T-cells, while proliferation of CD8^+^ T-cells was enhanced (154). Of note, in healthy donor-derived T-cells, no difference in the proliferation between PI3Kδ inhibited and control cells was observed (154). Phenotypically, an expansion of naïve and effector T-cells, accompanied by a reduction of memory cell populations (143, 154), and lower levels of PD-1 and TIM-3 expression of idelalisib-treated compared to untreated CAR T-cells of CLL patients were seen (154). This lower state of pre-activation resulted in an increased TNFα and IFNγ production *ex vivo* (154). And chromium release assays showed that cytotoxicity of idelalisib-treated CAR T-cells was not altered *in vitro* compared to untreated CAR T-cells (154). This data suggest that priming of CAR T-cells by idelalisib causes a less differentiated and activated phenotype of the T-cell product, which could result in an enhanced anti-tumoral activity *in vivo*. Investigating their function *in vivo*, two independent groups showed an enhanced tumor control and prolonged survival of PI3Kδ inhibitor pre-treated CAR T-cells in xenograft mouse models of CLL (143, 154) (**Figure 3**).

In conclusion, pre-treatment of cells for adoptive transfer therapies, such as CAR T-cells, with PI3Kδ inhibitors results in a less differentiated and activated cell product accompanied by an enhanced *in vivo* efficiency in xenograft models. Further studies are needed to assess the long-term persistence and tumor control of PI3Kδ inhibitor-primed CAR T-cells in an immunocompetent TME. Ultimately, a confirmation of these results in patients is still pending.

#### Effect of Phosphoinositide 3-Kinase α and β Inhibition on T-Cells

PI3Kδ is the most studied isoform in T-cells. To investigate the role of other PI3K subtypes in CD4^+^ as well as CD8^+^ T-cells, initially pan-class IA PI3K inhibitors were utilized. These studies showed a similar effect as p110δ isoform specific inhibition, such as reduced TCR signaling, proliferation, and higher expression of naïve/memory T-cell markers (137, 138). Intriguingly, neither the specific inhibition of PI3Kα nor -β did result in an altered TCR signaling, proliferation (137, 138), or affected the phenotype or cytokine production of CD8^+^ T-cells *in vitro* (138). This data suggest that neither PI3Kα nor -β alone are indispensable for T-cell function. To elucidate whether PI3Kα or -β inhibition have additive effects to PI3Kδ inhibition on T-cells, combinations of Class IA inhibitors and genetically inactive isoforms have been investigated. Treatment of PI3Kδ-deficient cells with PI3Kα or -β inhibitors further reduced TCR signaling as well as proliferation of CD4^+^ T-cells *in vitro* (137, 145) and *in vivo* (145). Concomitantly, higher frequencies of CD8^+^ T-cells with lower PD-1 expression at the tumor site have been noted upon PI3Kα and -δ inhibition (145). Of importance, all results were comparable to single δ-specific inhibition (145), and differences in T-cell phenotype as well as function have not been observed upon combination of PI3Kα and -β inhibition (137).

These results highlight that PI3Kδ is indispensable for T-cell activation and proliferation and its loss of function cannot be compensated by class IA PI3K isoforms. Dual inhibition of PI3Kα and -δ likely does not have additive effects, although a direct head to head comparison is missing.

#### Effect of Phosphoinositide 3-Kinase γ Inhibition on T-Cells

PI3Kγ inhibition is mostly targeting myeloid cells, as detailed above. Interestingly, an increased frequency of T-cells in tumor-bearing animals with either pharmacological or genetical PI3Kγ inhibition has been noted (96). To exclude bystander effects *via* the myeloid cell compartment, which is affected by dysfunctional PI3Kγ, myeloid cells of LLC-tumor bearing mice were depleted using clodronate liposomes and mice were treated with PI3Kγ inhibitors. In comparison to vehicle-treated animals, PI3Kγ inhibition did not result in altered T-cell numbers suggesting that it does not affect T-cell function markedly (96). In line, genetical or antibody-mediated depletion of CD8^+^ T-cells in PI3Kγ^−/−^ mice caused a reduced tumor control but no alterations in proliferation or production of IFNγ or GzmB of PI3Kγ-deficient T-cells isolated from either naïve or tumor bearing mice have been observed *ex vivo* (96). In conclusion, PI3Kγ inhibition is likely not affecting T-cell function directly. Nevertheless, the effects of PI3Kγ inhibition on other components of the TME, such as myeloid cells, can enhance the recruitment and anti-tumoral of T-cells.

## Resistance to Phosphoinositide 3-Kinase Inhibition in Cancer and Chronic Lymphocytic Leukemia

Although both isoform-specific and pan-PI3K inhibitors show promising clinical efficacy in certain human cancers, there are several intrinsic and acquired resistance mechanisms that challenge their use. The PI3K/AKT pathway involves numerous feedback loops and converges with other signaling pathways at several points. This crosstalk mechanism enables the tumors to adapt by directing the signaling to an alternative path and thereby influencing the therapeutic outcomes (155, 156). *RAS* oncogene that activates both RAF-MAPK and PI3K signaling is one of the striking examples. The two pathways converge at early points after the growth factor receptor stimulation and interestingly, they evolved as antagonists (156). It has been shown that inhibition of the PI3K pathway can cause over-activation of the RAF-MAPK pathway, which induces tumor growth and quenches the efficacy of PI3K inhibition (156). Dual inhibition strategies have strong potential to overcome this dilemma but likely to result in high toxicities and a narrow therapeutic window.

Presence of a genetic alteration that leads to over-activation of the targeted kinase usually predicts the success of the inhibition therapy, as proven in different malignancies (28). However, cancer cells are genetically complex and mutations downstream of the targeted node might also influence the cellular responses to inhibition resulting in reduced sensitivity. In line, activating mutations of the NOTCH pathway induced resistance to PI3K inhibition in breast cancer (157).

Besides pathway-related mutations, so-called “gatekeeper” mutations that deteriorate the sensitivity of tumor cells to the inhibitor can be acquired during the therapy. A common feature of these “gatekeeper” mutations is their presence in the kinase domain of the targeted protein kinase which hinders its binding to the inhibitor. Similar mutations in PI3K are likely to exist. However, studies analyzing the resistance mutations are scarce. Zunder et al. have detected a potential hotspot mutation in p110α that confers resistance by inhibiting the potency of several PI3K inhibitors 5- to 30-fold (158). To our knowledge, other PI3K isoforms have not been analyzed but these results might serve as a start point for the development of new generation, isoform selective PI3K inhibitors since the hotspot is conserved in the entire PI3K family (158). The nature of PI3K inhibition itself is likely to provide a supportive environment for new mutations to occur, as PI3K inhibition *in vitro* is not cytotoxic but mostly cytostatic for cancer cells (58, 159, 160). Upon inhibition, it has been shown that cells enter a dormant state and can survive with subtle amount of PI3K activity for a significant time (161) during which the accumulation of new mutations might be facilitated.

In CLL, treatment failure of idelalisib caused by *de-novo* mutations seems to be a rather rare event but disease progression is observed (162). Overall response rates (ORR) are in general relatively high in phase III trials of relapsed/refractory CLL patients with 83.6% for idelalisib/rituximab (median progression-free survival (PFS) 19.4 months) (129), and 70.0% for idelalisib/bendamustine + rituximab (median PFS 20.8 months) (163), but results of long-term follow-ups are still pending. Nevertheless, activating mutations of the BRAF-MAPK pathway have been observed in 10 non-responders to idelalisib (N=7) or voxtalisib (N=3), which targets the PI3K/mTOR pathway. Recurrent mutations were identified in *BRAF* (N=5), *MAP2K1* (N=2), and *KRAS* (N=2), but no information is provided whether these mutations resulted in resistance to idelalisib or voxtalisib (164). To investigate acquired resistance to idelalisib, samples of 13 CLL patients that initially responded to idelalisib treatment and developed progressive disease were analyzed by whole exome sequencing across three phase III trials (165). Intriguingly, neither recurrent, nor “gatekeeper” mutations or genomic alterations of any other related signaling pathways were identified that could confer resistance to idelalisib treatment. Similarly, in a preclinical mouse model, long-term PI3Kδ inhibition and serial tumor transplantations caused resistance to the treatment, but no recurrent mutations were identified by whole exome sequencing (166). Intriguingly, expression of insulin-like growth factor 1 receptor (IGF1R) was upregulated due to enhanced activity of forkhead box protein O1 (FOXO1) and glycogen synthase kinase 3 beta (GSK3β) which resulted in pronounced MAPK signaling pathway activity (166).

Despite all the challenges and resistance mechanisms, pharmacological targeting of the PI3K pathway is an efficient strategy. It is highly likely that long-term follow up data of CLL patients will provide more information about resistance mechanisms and combination strategies to achieve a durable effect with minimum toxicity.

## On-Target Side Effects of Phosphoinositide 3-Kinase Inhibition in Cancer and Chronic Lymphocytic Leukemia

As PI3Ks are involved in many different cellular functions and crucial signaling pathways, off-target effects need to be considered when treating patients, which has been extensively reviewed (167–169). In brief, inhibition of the α-isoform with alpelisib in breast cancer frequently caused hyperglycemia, rash, diarrhea or stomatitis (167). In CLL, PI3Kδ inhibition using idelalisib caused immune-cell mediated adverse events like pneumonitis, neutropenia, diarrhea, colitis or transaminitis, and an infiltration of T-cells to inflamed tissues has been observed. These adverse events were more frequent when combining idelalisib with rituximab-bendamustine or ofatumumab (129, 148, 168, 169). Recently, Kienle and Stilgenbauer raised optimism that i) the development of more specific PI3Kδ inhibitors like umbralisib, ii) the adaptation of the scheduling of PI3K inhibitors (e.g., intermittent dosing), and iii) combinatory treatment approaches could result in a more favorable toxicity profile and deep treatment responses with PI3K inhibitors (169).

## Conclusion

Initial success of idelalisib, the first approved PI3K-inhibitor for CLL, paved the development of a plethora of PI3K inhibitors, as recently reviewed by Kienle and Stilgenbauer (169). However already in 2016, concerns arose that clinical PI3Kδ inhibition can be associated with an increased frequency of immune-related adverse events (AE), such as colitis, pneumonitis, neutropenia as well as elevated hepatic transaminases (129, 168). This highlights the need for a detailed review of the expression as well as function of PI3Ks in components of the TME of CLL, which could serve as potential explanation for the observed AE during idelalisib treatment.

PI3Ks are involved in crucial signaling cascades of both, the non-immune and immune compartment of the TME in CLL. Some PI3K isoforms are expressed by all cell types whereas others are specific for distinct leukocyte subsets. The use of selective inhibitors gives the opportunity to enhance anti-tumoral immune responses of some cell types. An example is the effect of PI3Kγ inhibition on myeloid cells. Therefore, the challenge of subtype specific PI3K inhibition is not to dampen the function of other immune cells that are essential for tumor or pathogen control.

PI3Kδ-selective inhibition and its effect on the immune environment has so far been studied most in CLL. Apart its great success in controlling the CLL progression (129, 163, 170), adverse events, such as immune cell-mediated liver toxicities are a drawback. Reduced frequencies of circulating Tregs accompanied by an enhanced CD8^+^ T-cell infiltration to liver tissues have been reasoned to be involved in those cases (148). For Tregs, accumulating pre-clinical as well as clinical evidence supports that PI3Kδ inhibition reduces this population in numbers and dampens their immunosuppressive function. In contrast, the effect of PI3kδ inhibition on CD8^+^ T-cells is still under debate. On the one hand, diminished Treg-mediated immunosuppression was suggested to unleash CD8^+^ T-cell function, but on the other hand direct, negative effects of PI3Kδ inhibition on the function of this cell type have been observed. The latter could be causative for compromised clearance of pathogens, especially viruses, as well as a reduced anti-tumor control.

Neutrophils are important for the clearance of extracellular pathogens such as bacteria and fungi. Therefore, neutropenia, an AE observed in idelalisib-treated CLL patients, could be reasoned for higher rates of infections such as pneumonia. *In vitro* treatment of neutrophils with PI3Kδ inhibitors hindered their activation and their ADCC, supporting that inhibition of PI3Kδ in neutrophils could be involved in reduced immune function of idelalisib-treated CLL patients.

Today, little is known about the effect of other PI3K isoforms on the function of the TME in CLL. In contrast, many clinical trials investigating novel PI3Kδ inhibitors, dual inhibitors of PI3Kδ and -γ, as well as pan-Class IA inhibitors have been initiated (169). Therefore, thorough investigations of the TME in these trials are essential to elucidate the role of PI3K class I isoforms on the function of distinct cell types and to reassure that PI3K inhibitors can serve as a highly active, safe, and tolerable treatment option in CLL.

## Author Contributions

EA reviewed the literature, prepared the figures, wrote and revised the manuscript. SF, MSa, and LC reviewed the literature and prepared a draft of the manuscript. MSe reviewed the literature and revised the final version of the manuscript. PMR reviewed the literature, wrote and revised the manuscript. All authors contributed to the article and approved the submitted version.

## Conflict of Interest

The authors declare that the research was conducted in the absence of any commercial or financial relationships that could be construed as a potential conflict of interest.
